# Impact of COVID-19 on access to and delivery of sexual and reproductive healthcare services in countries with universal healthcare systems: A systematic review

**DOI:** 10.1371/journal.pone.0294744

**Published:** 2024-02-23

**Authors:** Michelle W. Tam, Victoria H. Davis, Monish Ahluwalia, Rachel S. Lee, Lori E. Ross

**Affiliations:** 1 University of Toronto Dalla Lana School of Public Health, Toronto, ON, Canada; 2 Upstream Lab, MAP Centre for Urban Health Solutions, Li Ka Shing Knowledge Institute, St. Michael’s Hospital, Toronto, ON, Canada; 3 Institute of Health Policy, Management and Evaluation, University of Toronto, Toronto, ON, Canada; 4 University of Toronto Faculty of Medicine, 1 King’s College Circle, Toronto, ON, Canada; University of Salamanca, SPAIN

## Abstract

**Objectives:**

The COVID-19 pandemic has caused unforeseen impacts on sexual and reproductive healthcare (SRH) services worldwide, and the nature and prevalence of these changes have not been extensively synthesized. We sought to synthesise reported outcomes on the impact of COVID-19 on SRH access and delivery in comparable countries with universal healthcare systems.

**Methods:**

Following PRISMA guidelines, we searched MEDLINE, Embase, PsycInfo, and CINAHL from January 1st, 2020 to June 6th, 2023. Original research was eligible for inclusion if the study reported on COVID-19 and SRH access and/or delivery. Twenty-eight OECD countries with comparable economies and universal healthcare systems were included. We extracted study characteristics, participant characteristics, study design, and outcome variables. The methodological quality of each article was assessed using the Quality Assessment with Diverse Studies (QuADS) tool. The Preferred Reporting Items for Systematic Reviews and Meta-analyses (PRISMA) guidelines were followed for reporting the results. This study was registered on PROSPERO (#CRD42021245596).

**Synthesis:**

Eighty-two studies met inclusion criteria. Findings were qualitatively synthesised into the domains of: antepartum care, intrapartum care, postpartum care, assisted reproductive technologies, abortion access, gynaecological care, sexual health services, and HIV care. Research was concentrated in relatively few countries. Access and delivery were negatively impacted by a variety of factors, including service disruptions, unclear communication regarding policy decisions, decreased timeliness of care, and fear of COVID-19 exposure. Across outpatient services, providers favoured models of care that avoided in-person appointments. Hospitals prioritized models of care that reduced time and number of people in hospital and aerosol-generating environments.

**Conclusions:**

Overall, studies demonstrated reduced access and delivery across most domains of SRH services during COVID-19. Variations in service restrictions and accommodations were heterogeneous within countries and between institutions. Future work should examine long-term impacts of COVID-19, underserved populations, and underrepresented countries.

## Introduction

Globally, the COVID-19 pandemic has exacerbated pre-existing inequities in accessing a range of healthcare services due to social distancing, lockdown measures, self-isolation, and clinic closures [1, 2]. Access to sexual and reproductive health (SRH) services including pregnancy care, safe abortion, contraception, treatment of sexually transmitted infections (STIs) and gynaecological malignancies, is critical to overall health and quality of life [3, 4]. During the COVID-19 pandemic, public health officials worldwide prioritised pandemic-related services over routine/elective healthcare services [5]. Lockdown conditions varied from Australia phasing out lockdowns in October 2021 and declaring emergency responses finished in September 2022 [6] to the United Kingdom implementing a final lockdown in January 2021 and all restrictions were lifted in February 2022 [7]. Thus, a wide variety of health delivery and health policy adaptations were implemented across jurisdictions for continued provision of SRH given pandemic restrictions. However, there is a paucity of current synthesised evidence on the impact of COVID-19 on access and delivery of SRH services. Understanding these changes allows us to examine the impact of COVID-19 on different aspects of SRH, which can guide current and future policy and institutional responses within and beyond the pandemic’s context. This systematic review aims to examine the impact of COVID-19 on access to and delivery of SRH services in countries with universal healthcare.

## Methods

This systematic review was prospectively registered with the International Prospective Register of Systematic Reviews (PROSPERO) database (Registration number CRD42021245596) and followed the Preferred Reporting Items for Systematic Reviews and Meta-Analyses (PRISMA) guidelines [8].

### Information sources and search strategy

Four databases were searched: MEDLINE, Embase, PsycINFO, and CINAHL. A literature search strategy was formulated with the assistance of an Information Specialist at the University of Toronto. Search terms relating to sexual health, reproductive health, contraception, fertility, pregnancy, and abortion were combined with COVID-19 (e.g., coronavirus) and health services access and delivery to identify relevant studies (see S1 Fig).

#### Study selection

Peer-reviewed French- and English-language original research publications from January 1, 2020 to June 6^th^, 2023 reporting on COVID-19 and SRH access and/or delivery in high-income Organisation for Economic Co-operation and Development (OECD) countries with comparable economies and universal healthcare (UHC) systems were included (see S1 Table) [9]. Countries included in this review are high-income countries as classified by the World Bank and must have universal [or near-universal] coverage for core medical services [9]. Implementation guidelines, reviews, commentaries, conference abstracts, meta-analyses, and editorial texts were excluded. Further, to focus on the impact of COVID-19 on SRH services, articles were excluded if they only described access or care delivery without either quantitative or qualitative comparison to a time period prior to the pandemic or prior experience.

Covidence systematic review software (Veritas Health Innovation, Melbourne, Australia) was used to manage and identify duplicates from the search results. Two independent reviewers (MWT, VHD, MA, and/or RL) screened titles and abstracts and then reviewed the full text to determine eligibility. Disagreements were resolved through consensus.

### Data extraction and synthesis of results

Two independent reviewers (MWT, VHD, and/or MA) extracted information on: authors, date of publication, country, study design, data collection dates, objectives, sample size, participant characteristics, inclusion and exclusion criteria, relevant outcome variables, and main findings.

Outcomes were categorised into eight sections: antepartum care, intrapartum care, postpartum care, assisted reproductive technology (ART) access, abortion access, gynaecological care, sexual health services, and human immunodeficiency virus (HIV) care.

### Quality appraisal assessment

A critical quality appraisal was conducted using the validated Quality Appraisal for Diverse Studies (QuADS) tool [10]. This tool was chosen due to the diversity of research design methodologies among the included studies. Two reviewers (VHD, MA) independently scored each article between 0–3 across thirteen questions, and a third reviewer (MWT) resolved discrepancies which were discussed and agreed upon during a meeting. The appraisal (S2 Table) informed our discussions about the research.

## Results

The search strategy (S1 Fig) yielded 3852 original database citations, of which 843 duplicates were removed. A total of 3009 titles and abstracts were screened. Of these, 241 underwent full-text screening, and 82 were included in the systematic review. Of the 28 UHC countries eligible for inclusion, 27 were represented in this review, as there were no studies from the Republic of Korea. The distribution of studies across countries is described in Table 1.

**Table 1:** Characteristics of studies included in this review (N = 82).

Study Location	#of Studies	Citation
Australia	16	[Chow et al., 2021] [73], [Homer et al., 2021] [41], [Traeger et al., 2021] [88], [Weerasuria et al., 2021] [85], [Trinh et al., 2022] [25], [Bradfield et al., 2022] [26], [Potenza et al., 2021] [27], [Sweet et al., 2021] [28], [Kluwgant et al., 2022] [38], [Wilson et al., 2022] [36], [Phillips et al., 2021] [74], [Coombe et al., 2021] [77], [Bittleston et al., 2022] [70], [Lee et al., 2021] [84], [Newman et al., 2022] [62], [Henry et al., 2022] [95]
Belgium	2	[De Kort et al., 2021] [56], [El Moussaoui et al., 2021] [87]
Canada	13	[Cameron et al., 2021] [53], [Hukku et al., 2022] [58], [Ennis et al., 2021] [57], [Lam et al., 2022] [65], [Wood et al., 2022] [81], [Gomez et al., 2021] [68] [Clark et al., 2023] [39], [Ryu et al., 2023] [75], [Boisvert et al., 2022] [43], [Souleymanov et al., 2023] [82], [Bayrampour & Tsui, 2023] [54], [Baaske et al., 2022] [71], [Khoury et al., 2022] [19]
Denmark	1	[Overbeck et al., 2020] [21]
France	3	[Doncarli et al., 2021] [23], [Atay et al., 2021] [60], [Mallaury et al., 2022] [59]
Germany	2	[Rød et al., 2023] [63], [Hertle et al., 2022] [34]
Ireland	2	[Greene et al., 2022] [61], [Schaler et al., 2022] [66]
Israel	4	[Justman et al., 2020] [24], [Binyamin et al., 2021] [50], [Karavani et al., 2021] [67], [Herzberger et al., 2022] [47]
Italy	6	[Dorizzi et al., 2021] [11], [Dell’Utri et al., 2020] [42], [Corrao et al., 2021] [13], [Cena et al., 2021] [12], [Brandell et al., 2021] [55], [Giacomelli et al., 2021] [83]
Japan	2	[Komatsu et al., 2020] [48], [Nakagawa et al., 2021] [37]
Multiple European Countries	1	[Ceulmans et al., 2021] [40]
The Netherlands	2	[Cui et al., 2023] [64], [Gamberini et al., 2023] [33]
New Zealand	2	[Rose et al., 2021] [79], [Dixon et al., 2023] [31]
Norway	1	[Baravelli et al., 2022] [20]
Slovenia	1	[Munda et al., 2022] [46]
Spain	4	[Gonzalez-Timoneda et al., 2021] [49], [Quirós-González et al., 2023] [86], [Cruz-Ramos et al., 2023] [32], [Suárez-Cortés et al., 2023] [96]
Sweden	1	[Zaigham et al., 2022] [44]
UK	19	[Harkness et al., 2021] [45], [Jardine et al., 2021] [22], [Leung et al., 2021] [72], [Lewis et al., 2021] [76], [Rimmer et al., 2020] [30], [Sarre et al., 2021] [16], [John et al., 2021] [17], [Riley et al., 2021] [29], [Fletcher et al., 2021] [35], [Silverio et al., 2021] [15], [Aydin et al., 2022] [51], [Bosó Pérez et al., 2022] [14], [Balachandren et al., 2022] [78], [Bekaert & Azzopardi, 2022] [80], [Lowe-Zinola et al., 2022] [69], [Moltrecht et al., 2022] [52], [Montgomery et al., 2023] [18], [Ma et al., 2022] [97], [Wilson et al., 2022] [36]
**Study Design**		
Cross-Sectional	33	[Jardine et al., 2021] [22], [Dorizzi et al., 2021] [11], [Homer et al., 2021] [41], [Ceulmans et al., 2021] [40], [Justman et al., 2020] [24], [Rimmer et al., 2020] [30], [Nakagawa et al., 2021] [37], [Overbeck et al., 2020] [21], [Harkness et al., 2021] [45], [Komatsu et al., 2020] [48], [Rose et al., 2021] [79], [Lewis et al., 2021] [76], [Weerasuria et al., 2021] [85], [Cena et al., 2021] [12], [Sarre et al., 2021] [16], [Doncarli et al., 2021] [23], [Fletcher et al., 2021] [35], [Kluwgant et al., 2022] [38], [Aydin et al., 2022] [51], [Wilson et al., 2021] [36], [Schaler et al., 2022] [66], [Phillips et al., 2021] [74], [Coombe et al., 2021] [77], [Bittleston et al., 2022] [70], [Wood et al., 2021] [81], [Karavani et al., 2021] [67] [Boisvert et al., 2022] [43], [Zaigham et al., 2022] [44], [Souleymanov et al., 2023] [82], [Ma et al., 2022] [97], [Khoury et al., 2022] [19], [Suárez-Cortés et al., 2023] [96], [Hertle et al., 2022] [34]
Observational	24	[Dell’Utri et al., 2020] [42], [Leung et al., 2021] [72], [Chow et al., 2021] [73], [Traeger et al., 2021] [88], [Corrao et al., 2021] [13], [Trinh et al., 2022] [25], [Potenza et al., 2021] [27], [Binyamin et al., 2021] [50], [Brandell et al., 2021] [55], [Greene et al., 2022] [61], [Lam et al., 2022] [65], [Balachandren et al., 2022] [78], [Bekaert & Azzopardi, 2021] [80], [Giacomelli et al., 2021] [83], [Lee et al., 2021] [84], [El Moussaoui et al., 2021] [87], [Gomez et al., 2021] [68] [Munda et al., 2023] [46], [Quirós-González et al., 2023] [86], [Lowe-Zinola et al., 2022] [69], [Baravelli et al., 2022] [20], [Herzberger et al., 2022] [47], [Mallaury et al., 2022] [59], [Baaske et al., 2022] [71],
Mixed Methods	9	[De Kort et al., 2021] [56], [Bradfield et al., 2021] [26], [Ennis et al., 2021] [57], [Atay et al., 2021] [60], [Rød et al., 2023] [63], [Clark et al., 2023] [39], [Cui et al., 2023] [64], [Henry et al., 2022] [95], [Wilson et al., 2022] [36]
Qualitative	16	[John et al., 2021] [17], [Riley et al., 2021] [29], [Silverio et al., 2021] [15], [Gonzalez-Timoneda et al., 2020] [49], [Cameron et al., 2021] [53], [Hukku et al., 2022] [58], [Bosó Pérez et al., 2022] [14], [Sweet et al., 2021] [28], [Ryu et al., 2023] [75], [Newman et al., 2022] [62], [Moltrecht et al., 2022] [52], [Bayrampour & Tsui, 2023] [54], [Montgomery et al., 2022] [18], [Dixon et al., 2023] [31], [Cruz-Ramos et al., 2023] [32], [Gamberini et al., 2023] [33]
**Theme**		
Antepartum Care	33	[Jardine et al., 2021] [22], [Dorizzi et al., 2021] [11], [Dell’Utri et al., 2020] [42], [Homer et al., 2021] [41], [Ceulmans et al., 2021] [40], [Justman et al., 2020] [24], [Rimmer et al., 2020] [30], [Nakagawa et al., 2021] [37], [Overbeck et al., 2020] [21], [Corrao et al., 2021] [13], [Bosó Pérez et al., 2022] [14], [Silverio et al., 2021] [15], [Cena et al., 2021] [12], [Sarre et al., 2021] [16], [John et al., 2021] [17], [Doncarli et al., 2021] [23], [Trinh et al., 2022] [25], [Bradfield et al., 2021] [26], [Potenza et al., 2021] [27], [Sweet et al., 2021] [28], [Riley et al., 2021] [29], [Fletcher et al., 2021] [35] [Clark et al, 2021] [39] [Munda et al., 2022] [46] [Baravelli et al., 2022] [20] [Boisvert et al., 2023] [43] [Zaighama et al., 2022] [44] [Montgomery et al., 2022] [18] [Wilson et al., 2022] [36] [Khoury et al., 2023] [19] [Cruz-Ramos et al., 2023] [32] [Gamberini et al., 2023] [33] [Hertle et al., 2022] [34]
Intrapartum Care	23	[Harkness et al., 2021] [45], [Jardine et al., 2021] [22], [Dorizzi et al., 2021] [11], [Dell’Utri et al., 2020] [42], [Homer et al., 2021] [41], [Justman et al., 2020] [24], [Rimmer et al., 2020] [30], [Komatsu et al., 2020] [48], [Kluwganta et al., 2022] [38], [Bradfield et al., 2021] [26], [Silverio et al., 2021] [15], [Cena et al., 2021] [12], [Binyamin et al., 2021] [50], [Gonzalez-Timoneda et al., 2020] [49], [Aydin et al., 2022] [51], [Sweet et al., 2021] [28], [Wilson et al., 2021] [36] [Munda et al., 2023] [46] [Haikin Herzberger et al., 2022] [47] [Dixon et al., 2023] [31] [Boisvert et al., 2023] [43] [Zaigham et al., 2022] [44] [Cruz-Ramos et al., 2023] [32]
Postpartum Care	13	[Jardine et al., 2021] [22], [Ceulmans et al., 2021] [40], [Bradfield et al., 2021] [26], [Silverio et al., 2021] [15], [Riley et al., 2021] [29], [John et al., 2021] [17], [Cameron et al., 2021] [53] [Sweet et al., 2021] [28] [Moltrecht et al., 2022] [52] [Boisvert, et al., 2022] [43] [Bayrampour et al., 2023] [54] [Khoury et al., 2022] [19] [Dixon et al., 2022] [31]
Abortion	11	[De Kort et al., 2021] [56], [Brandell et al., 2021] [55], [Greene et al., 2022] [61], [Hukku et al., 2022] [58], [Ennis et al., 2021] [57], [Cena et al., 2021] [12], [Atay et al., 2021] [60], [Rød et al., 2023] [63], [Cui et al., 2022] [64], [Newman et al., 2022] [62] [Mallaury et al., 2022] [59]
Assisted Reproductive Technologies	5	[Komatsu et al., 2020] [48], [Bosó Pérez et al., 2022] [14], [Schaler et al., 2022] [66], [Lam et al., 2021] [65] [Karavani et al., 2021] [67]
Gynecology	8	[Dell’Utri et al., 2020] [42], [Rimmer et al., 2020] [30], [Komatsu et al., 2020] [48], [Leung et al., 2021] [72], [Gomez et al., 2021] [68], [Bosó Pérez et al., 2021] [14], [Bittleston et al., 2022] [70] [Lowe-Zinola et al., 2022] [69] [Newman et al., 2022] [62] [Baaske et al., 2022] [71]
Sexual Health	16	[Chow et al., 2021] [73], [Rose et al., 2021] [79], [Bosó Pérez et al., 2022] [14], [Balachandren et al., 2021] [78], [Phillips et al., 2021] [74], [Coombe et al., 2021] [77], [Ennis et al., 2021] [57], [Cena et al., 2021] [12], [Bekaert & Azzopardi, 2021] [80], [Bittleston et al., 2022] [70], [Wood et al., 2021] [81] [Lewis et al., 2021] [76] [Newman et al. 2022] [62] [Ryu et al., 2023] [75] [Baaske et al., 2022] [71] [Souleymanov et al., 2023] [82]
HIV Care	10	[Traeger et al., 2021] [88], [Weerasuria et al., 2021] [85], [Phillips et al., 2021] [74], [Giacomelli et al., 2021] [83], [Lee et al., 2021] [84], [El Moussaoui et al., 2021] [87], [Wood et al., 2021] [81] [Newman et al., 2022] [62] [Souleymanov et al., 2023] [82] [Quirós-Gonzáleza et al., 2023] [86]

Table 2 provides a summary of the main results of the studies and the QuADS results. On average, studies had a quality score of 66.6% (25.97/39) across the 13 questions. The quality appraisal assessment found that studies typically scored high (2–3) on the research aims statement, description of research setting and target population, the appropriateness of their study design, and conceptual underpinnings to the research. On average, studies had the lowest scores on their involvement of research stakeholders in the research process, followed by their justification of the analytic method and discussion of strengths and limitations.

**Table 2:** Summary of main results of the studies included in this review (N = 82).

Authors	Country	Sample Size	Participant Characteristics	Study Design	Sampling Strategy	Main Outcome Variables	QuADS
Atay et al, 2021 [60]	France	809	Pregnant women	Mixed methods	Convenience	• Changes in telemedicine abortion service requests at different periods of lockdown • Restrictions to accessing abortion care during pandemic	27
Aydin et al., 2022 [51]	England	477	Expecting parents	Mixed methods	Convenience	• Changes to birth and birth plan due to COVID-19	30
Baaske et al., 2022 [71]	Canada	3691	Women aged 25–69	Observational	Consecutive clinic	• Access to contraception • Participation in cervical and breast cancer screening	21
Balachandren et al., 2022 [78]	United Kingdom	9784	Pregnant women	Observational	Population	• Access to contraception and pregnancy intentions	31
Baravelli et al., 2022 [20]	Norway	1 244 560	Female residents of Norway age 15–50	Observational	Population	• Pregnancy-related inpatient, outpatient, and primary care health utilisation before, during, and after COVID-19 restrictions	32
Bayrampour & Tsui, 2023 [54]	Canada	268	Pregnant adults living in British Columbia	Cross-sectional	Purposive	• Access to postpartum healthcare services	28
Bekaert et al., 2021 [80]	United Kingdom	Varies across variables	Youth aged 13–17	Cross-sectional	Consecutive clinic	• Changes in the number of consultations before and during the pandemic	19
Binyamin et al., 2021 [50]	Israel	413	Pregnant women receiving caesarean delivery	Observational	Consecutive clinic	• Differences between rates of neuraxial anaesthesia before and during COVID-19 pandemic	25
Bittleston et al., 2022 [70]	Australia	1056	Predominantly female young adults (16–29 years old)	Cross-sectional	Convenience	• Access to sexual health services	25
Boisvert et al., 2022 [43]	Canada	216	Women ≥ 16 years old who delivered during the first three months of the pandemic	Cross-sectional	Convenience	• Perceived quality of prenatal care, intrapartum care, and postpartum care	26
Bosó Pérez et al., 2022 [14]	United Kingdom	20	14 women, 6 men	Qualitative	Purposive	• Themes related to unmet access to SRH during COVID-19	30
Bradfield et al., 2022 [26]	Australia	620 surveys, 20 interviews	Registered midwives who provided care since March 2020	Mixed methods	Convenience	• The experience of midwives providing care during COVID-19	32
Brandell et al., 2021 [55]	Italy	778 requests	Women seeking medical abortion through telemedicine service	Observational study	Convenience	• Changes in need/requests for telemedicine abortion prior to and during the pandemic	20
Cena et al., 2021 [12]	Italy	77	Italian perinatal healthcare facilities	Cross-sectional	Convenience	• Impact on the activities and services provided by perinatal health facilities due to COVID-19	32
Ceulemans et al., 2021 [40]	Belgium, Norway, Netherlands, Switzerland, Ireland, United Kingdom	16063	Pregnant and breastfeeding women; most between 31–35 years old	Cross-sectional	Convenience	• Impact of COVID-19 on: 1) pregnancy experience; 2) breastfeeding practices & support; 3) access to health services	27
Chow et al., 2020 [73]	Australia	21576 consultations before and during lockdown	Men, women, and other individuals	Observational	Consecutive clinic	• Number of consultations (total) and for each STI diagnosis pre-lockdown, lockdown, and post-lockdown • Number of consultations for asymptomatic screening and sex work certificates • Time from symptom onset to presentation • Number of sexual partners reported in each time period	32
Clark et al., 2023 [39]	Canada	85 surveys, 6 interviews	Women with GDM who delivered at a tertiary care centre	Mixed methods	Consecutive clinic and convenience	• Impact of virtual care on gestational diabetes-related outcomes, patient experience, and healthcare provider experience	31
Coombe et al., 2021 [77]	Australia	518	Females ≤50 years old	Mixed-methods	Convenience	• Impact of COVID-19 on sexual and reproductive health services, including access to SRH provider, contraception use and access, and of general SRH services.	28
Corrao et al., 2021 [13]	Italy	52312	Pregnant women who delivered in-hospital	Observational	Consecutive clinic	• Proportion of women who had at least a gynaecology visit within 21d of delivery	26
Cruz-Ramos et al., 2023 [32]	Spain	14	Spanish women who delivered during the COVID-19 state of emergency	Qualitative	Purposive	• Prenatal, intrapartum, and postpartum experiences and expectations	26
Cui et al., 2023 [64]	The Netherlands	178	Women aged 19–40 who accessed Women on Web (WoW) for telemedicine abortion	Mixed methods	Convenience	• Accessibility of abortion services during the COVID-19 pandemic	21
De Kort et al., 2021 [56]	Belgium	11	Abortion centre clinic coordinator (1 female), psychosocial staff (7 female), physicians (2 female, 1 male)	Qualitative	Purposive	• Differences in the prevalence of abortion requests and actual abortions during lockdown • Procedure changes at the abortion clinic Influence of COVID-19 protective measures on staff and clients	24
Dell’Utri et al., 2020 [42]	Italy	Pre-COVID-19: 5644 admissions; during pandemic: 3647 admissions	Women of all ages and nationalities	Observational	Consecutive clinic	• Admission rates in obstetrics and gynaecology emergency services	29
Dixon et al., 2023 [31]	New Zealand	17	Women living in Aotearoa, New Zealand	Qualitative	Purposive	• Prenatal, intrapartum, and postpartum experiences	24
Doncarli et al., 2021 [23]	France	500	Women pregnant between March 17th and May 11th, 2020	Cross-sectional	Quota	• The impacts of the first lockdown in France on the frequency of voluntary changes in pregnancy monitoring and associated factors	26
Dorizzi et al., 2021 [11]	Italy	104	Pregnant women, mean age 32.5	Cross-sectional	Convenience	• Major worries during pregnancy and while in hospital • Access to prenatal services / online courses • Patient perception of safety and quality of service	29
El Moussaoui et al., 2021 [87]	Belgium	Varies across variables	People living with HIV	Observational	Consecutive clinic	• Number of new HIV diagnoses, medical care delays • Testing and HIV co-infections and comorbidities	18
Ennis et al., 2021 [57]	Canada	307 (first) and 78 (second)	Abortion health care professionals, including physicians, nurse practitioners, pharmacists and administrators	Mixed methods	Convenience	• Impact of pandemic on access to abortion car • Impact of pandemic on change of abortion practice and delivery	24
Fletcher et al., 2021 [35]	United Kingdom	251	Obstetricians	Cross-sectional	Convenience	• Views of obstetricians regarding self-monitoring for hypertensive disorders of pregnancy during COVID-19	25
Gamberini et al., 2023 [33]	The Netherlands	9 documents; 20 interviews	Documents describing antenatal care services; midwives and gynaecologists working in antenatal care	Qualitative	Purposive	• Guideline recommendations for antenatal care adaptations Impact of COVID-19 on healthcare providers and healthcare delivery practices	32
Giacomelli et al., 2021 [83]	Italy	70,349 HIV-RNA viral load determinations	Patients living with HIV attending outpatient clinic	Observational	Consecutive clinic	• Yearly HIV-RNA viral suppression rates and determinations • Medication collection and virological appointment attendance before and during COVID-19 • Trends of HIV-RNA determinations ≥ 50 copies/mL before and during COVID-19	29
Gomez et al., 2021 [68]	Canada	39,691	Patients presenting to the emergency department with appendicitis, cholecystitis, ectopic pregnancy, or miscarriage	Observational	Consecutive clinic	• Weekly rates of visits to the ED during the COVID-19 pandemic for certain presentations	29
Gonzalez-Timoneda et al., 2020 [49]	Spain	14	Midwives	Qualitative	Purposive	• Challenges and differences with working as a midwife in a pandemic	30
Greene et al., 2022 [61]	Ireland	764 requests for online telemedicine abortion care, 225 completed	Pregnant women seeking or interested in abortion	Mixed methods	Convenience	• Impact of COVID-19 restrictions on telemedicine abortion requests among patients seeking Women on Web service	32
Harkness et al., 2021 [45]	United Kingdom	92 United KingdomBoards and Trusts	Senior obstetricians and midwives	Cross-sectional	Convenience	• Changes in induction of labour (IOL) rates and practice • Impact on home cervical ripening • Women’s responses to IOL as reported by staff	23
Henry et al., 2022 [95]	Australia	109 s, 17 interviews	Healthcare providers of pregnancy and birth care	Mixed methods	Convenience	• Impact of COVID-19 on delivery, timeliness, and quality of pregnancy care	30
Hertle et al., 2022 [34]	Germany	1821 women; 1551 midwives	Women ≥ 18 years old who gave birth between May-November 2020; Midwives working in Germany in 2020	Cross-sectional	Convenience	• Impact of COVID-19 on the delivery of prenatal care by midwives	27
Herzberger et al., 2022 [47]	Israel	5563	Women admitted for delivery	Observational	Consecutive clinic	• Intrapartum maternal and perinatal outcomes	25
Homer et al., 2021 [41]	Australia	103	Privately practising midwives	Cross-sectional	Convenience	• Changes in demand for home births • Obtaining information on COVID-19 • Preparation and planning for COVID-19 • Altering practice to accommodate COVID-19 precautions	26
Hukku et al., 2022 [58]	Canada	23	Participants identifying as “women” and who have had an abortion after March 2020	Qualitative	Purposive	• Impact of COVID-19 on deciding to get an abortion and the type of abortion	23
Jardine et al., 2021 [22]	United Kingdom	81	Obstetric units in the United Kingdom	Cross-sectional	Convenience	• Service modifications to maternity services made during the pandemic.	29
John et al., 2021 [17]	United Kingdom	16	Pregnant and postpartum women belonging to ethnic minority communities	Qualitative	Purposive	• Themes related to women’s experience in maternity care during COVID-19	29
Justman et al., 2020 [24]	Israel	1352	Pregnant women, mean age 30.6, registered at the clinic	Cross-sectional	Consecutive clinic	• Changes in outpatient clinic visits during the COVID-19 outbreak • Changes in the rates of caesarean and instrumental deliveries during the COVID-19 outbreak • Changes in neonatal and maternal outcomes during the COVID-19 outbreak	27
Karavani et al., 2021 [67]	Israel	90	IVF clinic appointments	Cross-sectional	Consecutive clinic	• Impact of COVID-19 on tertiary fertility service	
Khoury et al., 2022 [19]	Canada	265	Women ≤ 36 weeks gestation ≥ 18 years old	Cross-sectional	Convenience	• Prenatal care and intrapartum outcomes Access to prenatal care	25
Kluwgant et al., 2022 [38]	Australia	1676	Women who delivered and midwives who worked during the COVID-19 pandemic	Cross-sectional	Convenience	• Experiences of pregnant women seeking sexual and reproductive health services during COVID-19 and factors related to their care and services	32
Komatsu et al., 2020 [48]	Japan	2446	Members of Japanese Society of Obstetrics and Gynecology (JSOG)	Cross-sectional	Convenience	• Changes in number of patients (pregnant women, operations for gynaecological malignancies and benign gynaecological conditions, treatments for gynaecological malignancy and reproductive technologies) • Prevalence of countermeasures to COVID-19 (including screening policy) • Delivery modes in COVID-19 positive pregnant women	26
Lam et al., 2022 [65]	Canada	NA	NA	Observational	Consecutive clinical	• Impact of COVID-19 on the availability and access to fertility services. Procedural volume of fertility services, including in vitro fertilisation and intracytoplasmic sperm injections, frozen embryo transfers, intrauterine and donor inseminations.	14
Lee et al., 2021 [84]	Australia	4551	People living with HIV	Cross-sectional	Consecutive clinical	• Impact of lockdown on access to antiretroviral therapy, including proportion of antiretroviral therapy provided to patients through mail access, and number of patients with controlled viral load	25
Leung et al., 2020 [72]	United Kingdom	585	Operations on patients who received surgery for gynecological cancer	Observational	Consecutive clinical	• Changes to the surgical workflow due to COVID-19 Differences in the surgical procedures performed between the time periods Intraoperative and early post-operative outcomes Impact on staff wellbeing and training	30
Lewis et al., 2021 [76]	Scotland	2005	Young adults [16–24] living in Scotland	Cross-sectional	Convenience	• Impacts of pandemic mitigation measures on young people’s access to, and use of, condoms and contraception.	29
Lowe-Zinola et al., 2022 [69]	United Kingdom	1943	Women with gynaecological cancer at a tertiary cancer centre	Observational	Consecutive clinical	• Decision alterations from standard of care due to COVID-19 Outcomes of patients whose decisions altered from the standard of care	27
Ma et al., 2022 [97]	United Kingdom	214	Women aged 16–54 seeking or using contraception during the first COVID-19 lockdown	Cross-sectional	Convenience	• Access to contraception during the COVID-19 lockdown	21
Mallaury et al., 2022 [59]	France	44 898	Women who had an abortion	Observational	Population	• Abortion-related clinical outcomes	24
Moltrecht et al., 2022 [52]	United Kingdom	17	Healthcare providers of perinatal care services	Qualitative	Purposive	• Healthcare providers’ perceptions of the needs of young parents during COVID-19 and the impact of COVID-19 on perinatal care delivery	28
Montgomery et al., 2022 [18]	United Kingdom	23	Women who received maternity care in South London	Qualitative	Purposive	• Impact of COVID-19 on the psychosocial experiences of pregnancy and maternal care	28
Munda et al., 2022 [46]	Slovenia	847	Women who received a GDM diagnosis during the pre-COVID-19 and COVID-19 periods	Observational	Consecutive clinic	• Glycemic control, weight gain, maternal outcomes, and perinatal outcomes	26
Nakagawa et al., 2021 [37]	Japan	77	Pregnant women	Cross-sectional	Convenience	• Acceptability of telemedicine by pregnant women • Experiences with video call system and telemedicine consultation for prenatal check-ups • Experience with mobile CTG device and associated costs for prenatal check-ups	24
Newman et al., 2022 [62]	Australia	15	Healthcare workers in public sexual health services	Qualitative	Purposive	• Adaptations in sexual and reproductive health care during COVID-19 • Impact of these adaptations on quality of care	18
Overbeck et al., 2020 [21]	Denmark	257	Pregnant women	Cross-sectional	Convenience	• Cancellation of scheduled antenatal appointments with general practitioners and midwives • Concerns about difficulty in accessing healthcare and factors that could improve care	23
Phillips et al., 2021 [74]	Australia	20	Directors of public sexual health clinics	Cross-sectional	Convenience	• Changes to service operations, patient intake and consultations, including for high-risk populations • Changes in STI sample testing and collection • Changes to service due to COVID-19 measures	27
Potenza et al., 2021 [27]	Australia	2,882,966	Antenatal attendances from January 2018 to April 2021	Observational	Consecutive clinical	• Trends in maternity care consultations and uptake of telehealth	16
Quiros-Gonzalez et al., 2023 [86]	Spain	2760	People with HIV who attended an HIV infection unit from 2016–2020	Observational	Consecutive clinical	• Impact of COVID-19 on clinical outcomes, emergency room visits or hospital admissions, and HIV disease control	26
Riley et al., 2021 [29]	United Kingdom	25 interviews	Pregnant women	Qualitative	Purposive	• The impact of COVID-19 restrictions on the antenatal and postpartum experience	22
Rimmer et al., 2020 [30]	United Kingdom	148	NHS units	Cross-sectional	Convenience	• Changes in maternity services Changes to gynaecology services Training specific to COVID-19	23
Rød et al., 2023 [63]	Germany	2057 s; 8 interviews	Women requesting telemedicine abortion; healthcare professionals providing abortion services	Mixed methods	Convenience	• Reasons for choosing telemedicine Perceptions of healthcare providers on access to abortion during COVID-19	29
Rose et al., 2021 [79]	New Zealand	500	15–24 year olds	Cross-sectional	Convenience	• Current and future sexual healthcare needs Telehealth consultation experience	29
Ryu et al., 2023 [75]	Canada	18	Sexual health service providers	Qualitative	Purposive	• Impact of COVID-19 on access to sexually transmitted infections and adaptations to care	27
Sarre et al., 2021 [16]	United Kingdom	60	Women in the 2nd or 3rd trimester with diabetes or gestational diabetes	Cross-sectional	Convenience	• Experience of women using antenatal diabetes services during COVID-19	25
Schaler et al., 2022 [66]	Ireland	135	Men and women scheduled for appointments at private not-for-profit fertility clinic	Cross-sectional	Convenience	• Disruption to fertility services	20
Souleymanov et al., 2023 [82]	Canada	366	Men ≥ 18 years old living in Manitoba who identify as gay, bisexual, queer, or two-spirit	Cross-sectional	Convenience	• Access to HIV testing Condom use practices	31
Silverio et al., 2021 [15]	United Kingdom	23	Women who delivered from March-August 2020	Qualitative	Purposive	• Changes experienced to care delivery during COVID-19	31
Stirling Cameron et al., 2021 [53]	Canada	8	Postpartum, Syrian refugee women living in Nova Scotia, Canada, with a child under 12 months old	Qualitative	Purposive	• Impact of COVID-19 postpartum and pregnancy experience	25
Suárez-Cortés et al., 2023 [96]	Spain	434	Women ≥ 18 years old who gave birth in the Murcia region	Cross-sectional	Convenience	• Changes to postpartum care delivery	28
Sweet et al., 2021 [28]	Australia	27	Pregnant and postpartum women	Qualitative	Purposive	• The experiences of women receiving maternity care during COVID-19	17
Traeger et al., 2021 [88]	Australia	19876	Gay and bisexual men	Observational	Consecutive clinical	• Number of PrEP prescriptions per week	27
Trinh et al., 2022 [25]	Australia	24,650	Women who delivered in the Local Health District of New South Wales	Observational	Population	• The impact of COVID-19 on changes in the use of telehealth	22
Weerasuria et al., 2021 [85]	Australia	153	People living with HIV	Cross-sectional	Convenience	• Access to antiretroviral therapy for HIV	20
Wilson et al., 2022 [36]	Australia	3364	Women who are currently pregnant or recently given birth	Cross-sectional	Convenience	• Changes to birth plan due to pandemic Changes to antenatal care delivery due to COVID-19 Maternity experience	22
Wilson et al., 2022 [36]	United Kingdom	45 maternity units; 166 pregnant women; 23 interviews	Maternity units; pregnant women; postpartum women	Mixed methods	Convenience	• Use of and perspectives on self-monitored blood pressure Perceived experiences of maternity care provision	24
Wood et al., 2022 [81]	Canada	1504	University students between 18–24 years old	Cross-sectional	Convenience	• Perceived impact of COVID-19 on access to sexual health services	31
Zaigham et al., 2022 [44]	Sweden	5003	Women who gave birth in Sweden	Cross-sectional	Convenience	• Maternal outcomes and perceived quality of prenatal and intrapartum care	24

### Antepartum care

Thirty-three studies reported the impact of COVID-19 on antepartum care. With regards to antepartum care access, a hospital in Italy noted that 20% of patients were unable to carry out all antepartum appointments due to service suspension, unavailability, or personal choice [11]. In fact, 23.4% of Italian peripartum facilities were partially or fully converted to COVID-19 units from March-May 2020 [12]. Data from the Italian National Health Service showed that 81% [compared to 99% in a reference cohort] of patients received an antepartum visit within 21 days prior to delivery during periods with strong restrictions [13]. Patients in the United Kingdom (UK), Canada, and Italy reported increased difficulty accessing antepartum care due to frequently re-scheduled or cancelled appointments [12, 14–19]. Decreased outpatient (17%) and primary care (10%) antepartum consultations were seen nationwide in Norway from March to April 2020 [20].However, in Denmark, a survey of pregnant women indicated very few patients had antepartum consultations cancelled by their general practitioner (GP) or midwife due to COVID-19 [21]. With regards to screening services, 15% of UK National Health Service (NHS) units reported decreased screening for fetal anomalies; over 25% of women forewent ultrasounds, genetic screening, or toxoplasmosis screening in France due to COVID-19-related reasons; and a tertiary medical centre in Israel noted visits for ultrasounds decreased by 18% during the lockdown period compared to the previous year [22–24].

To address reduced access, alternative modes of service delivery were introduced. For example, physicians and midwives in the UK, New Zealand, Spain, the Netherlands, Germany, Canada, and Australia reported increased virtual and telephone consultations compared to before the pandemic [17–19, 22, 25–34]. Additionally, nearly 80% of UK NHS units switched to home blood pressure monitoring and 32–53% switched to home urine testing with obstetricians in both the UK and Australia recommending home blood pressure and weight self-monitoring [22, 28, 35, 36]. In a tertiary hospital in Japan, women were provided the option for home blood pressure and fetal heart rate monitoring [37].

In Andalusia, Spain, women reported self-swabbing for group B streptococcus screening [32]. Gestational diabetes protocols were altered n Australia [38], Canada [39], and the UK [16] switching to virtual monitoring and/or relying on solely fasting glucose concentrations for diagnosis. Few countries, including Slovenia and Italy, maintained traditional gestational diabetes screening protocols [46]. Many institutions in the UK, France, Italy, New Zealand, the Netherlands, Spain, Canada, and Australia implemented measures to decreased viral spread during in-person appointments including social distancing, equipment sanitization, limiting support persons during visits, and increased personal protective equipment [12, 16, 19, 23, 28, 29, 31–33, 40, 41].

Additionally, many institutions noted decreased admissions from the emergency room for antepartum patients, including 86% of NHS units [22]. In Milan, admissions for bleeding in pregnancy decreased by 46.6% and gestational diabetes by 47.1% [though admissions for pregnancy-induced hypertension increased by 2.1%] [42]. At an Israel tertiary medical centre, visits to the high-risk clinic decreased by 32.8% compared to the prior year [24]. In Norway, inpatient admissions were reduced by 9% nationwide [20].

At tertiary hospitals in Canada and Sweden, approximately 50% of surveyed patients reported COVID-19 negatively impacted the quality of their prenatal care [43, 44].

### Intrapartum care

Twenty-three examined the impact of COVID-19 on intrapartum care. There were mixed results on the impact of COVID-19 on induction of labour [IOL], including the mode of delivery and location of birth. Three studies found no or little change to the number of inductions performed [24, 45, 46]. However, in the UK, 23% of Trusts and Boards switched to mechanical induction exclusively as it was perceived as safer for home use [45]. Further, 17% of UK maternity units temporarily paused some indications for IOL [22], and some UK practices ceased or reduced IOL [30]. Forty-two percent of clinics transformed their induction services to reduce hospitalizations or time in hospital [30]. Midwives working in Australian hospitals reported varying experiences with some reporting decreased IOL frequency [26]. However, studies in Italy [42] and Israel [47] reported slightly increased IOL rates during the pandemic compared to pre-pandemic.

There were reported changes in vaginal and caesarean births. An Israel-based study found increased operative vaginal births among nulliparous women due to less prepartum care and more high-risk pregnancies during the pandemic [24]. With regards to caesarean births, there was a spectrum of responses. An Italian study found increased elective caesarean births, aligned with shifts to planned labours to decrease time in hospital [42]. Some practices in the UK offered more opportunities for elective caesarean section, while others stopped or limited opportunities [30]. Some midwives in Australia reported fewer caesarean sections while others reported no change [26], and there were no significant differences in caesarean births among women diagnosed with gestational diabetes before and during COVID-19 in Slovenia [46] Some COVID-19 positive women in Japan had mandatory caesarean sections due to the likelihood of transmitting COVID-19 during labour due to heavy breathing [48]. In the UK, most facilities had a designated COVID-19 operating theatre for COVID-19 positive women [30]. Similarly, in Spain, a study reported that COVID-19 positive women were set up in specific birth rooms [49]. One study in Israel reported increased neuraxial anaesthesia rates instead of general anaesthesia for elective caesarean births [50].

Additionally, there was increased interest in home labour and delivery services due to restrictions on having loved ones present during labour, fear of contracting COVID-19, and restricted hospital birthing options [11, 26, 31, 38, 45]. For example, in an Australian study, 93% of privately practising midwives reported patients having greater interest in home births [41]. In addition, 28% of UK trusts and boards reported increased numbers of women returning home for cervical ripening [45]. Some midwives in Australia and the UK allowed loved ones to be present during labour, but limited the number that could attend during delivery [26, 41]. However, while women in Australia and the UK denied any significant changes in birth plans due to COVID-19 [36, 51], there were still meaningful differences such as reported prohibitions on water or home birth in the UK [22, 51]. For example, a UK study found that 59% of obstetric units prohibited home or freestanding midwife-led births and 32% changed their provision of water birth [22].

A few studies reported on quality of care during labour. In a Canadian study, 59% of women reported that COVID-19 did not affect their quality of care during labour, although 19% felt unfavourably about PPE requirements during labour and that it affected their birthing experience [43]. A Swedish study reported that half of women who had a caesarean section and 47% of women who went into labour reported that COVID-19 resulted in reduced overall quality of care [44].

### Postpartum care

Thirteen studies reported on the impact of COVID-19 on postpartum care. Studies in the UK, New Zealand, Canada, and Australia reported substantial and heterogeneous maternity service modifications, including reductions in postnatal appointments, additions of postpartum clinics in community settings, and changes from in-person to telephone and videoconferencing appointments [15, 22, 26, 29, 31, 43]. Two studies in the UK and one in Canada reported that young parents [52], women [15] and specifically, ethnic minority women [17], and refugee women [53] found that virtual care was inaccessible, not as effective, and inappropriate for postnatal care. A Canadian study found that 68.52% of patients reported COVID-19 negatively impacted postpartum care [43]. Meanwhile, a minority of obstetric units in the UK reported having no change in postpartum care [22].

Multinational studies reported that the pandemic adversely impacted access to postpartum services and supports, notably breastfeeding support, new parent classes, and in-home postpartum visits [19, 28, 29, 40, 54]. For example, compared to before COVID-19, less medical follow-up was reported from perinatal organisations, lactation consultants, general practitioners, and midwives [19, 40]. Insufficient support from healthcare professionals on breastfeeding was noted as one of the main reasons for breastfeeding cessation [40]. Studies in Canada, Australia, New Zealand, and the UK reported limitations on in-hospital support people during postpartum care [15, 26, 31, 52–54].

### Abortion

Twelve studies reported on the impact of COVID-19 on abortion access. There is some evidence that demand for abortion may have been impacted by COVID-19 [12, 55–57]. Varied levels of access and policy responses were reported, even within countries. One study across Canada found varying results where many abortion providers reported that the pandemic did not impact their ability to provide access, while some abortion providers reported barriers to timely care, fewer requests for abortion, and fewer operating theatres available [57]. Another Canadian study reported that COVID-19 exacerbated existing barriers to abortion care, especially with regards to the timeliness of multiple in-person appointments; however, all study participants were able to successfully access abortion care during the pandemic [58]. A French study reported decrease in total number of abortion, but increase in number of medical abortions for adapted access [59].

There was an increase in number of medical abortions provided via telehealth [55, 56, 60–64]. A study in the Netherlands reported that 37% of participants accessed telemedicine for abortion due to COVID-19 and was correlated with not living in a city with an abortion clinic [64].Similarly, a study in Germany reported that 38.8% of participants accessed telemedicine for abortion due to COVID-19 and the factors that impacted access included socioeconomic status, disability, language, place of residence, caring responsibilities, home environment, and refugee and immigrant status [63]. One study in Belgium showed decreased abortion requests during the pandemic, as well as a decreased number of abortions performed [56]. However, studies in Italy [55], France [60], and Ireland [61] showed an increase in the raw number of telemedicine abortion requests during the pandemic [55], and many participants reported that COVID-19 was the reason for consulting the telemedicine service [60, 61].

### Assisted reproductive technology [ART]

Five studies reported on the impact of COVID-19 on access to ART. International studies found that ART treatments decreased during the pandemic and were either postponed or cancelled [48, 65]. A study conducted in Ireland at a fertility clinic reported that over 85% of participants experienced disruptions in their fertility journey due to the initial COVID-19 lockdown (March–May 2020) [66]. A study at a tertiary teaching hospital reported that all new IVF cycles were cancelled in March 2020 and all existing appointments were given alternatives [67]. As a result, 37.8% of patients continued with in-person appointments, while 30% chose telemedicine appointments, and 32.2% of patients cancelled their appointments [67]. In Japan, 74.3% of facilities that provide treatments in reproductive technologies reported decrease in treatments [48].

### Gynaecology

Ten studies reported the impact of COVID-19 on gynaecological services. Overall, studies highlighted the suspension of elective gynaecological services and reduced surgical and oncology capacity due to the pandemic. In the UK, 88.5% of NHS units suspended elective urogynaecology services and 43.9% instituted protocols to avoid emergency laparotomy in women with suspected or confirmed COVID-19 [30]. Many NHS units instituted medical management of miscarriage and confirmed ectopic pregnancy as first-line treatment to reduce inpatient stays, however, a study in Canada reported no changes in the frequency of medical or surgical management [30, 68]. A study in Japan noted decreased surgeries for benign gynaecological conditions in 2020 vs. 2019 [48]. A study in Italy noted significantly decreased admissions for gynaecological complaints in 2020 vs. 2019 [42]. In Canada, there was a sustained decrease in weekly ER visits for miscarriage with no increased demand after lockdowns were lifted, suggesting that miscarriage management may have been shifted to outpatient clinics [68].

Studies in the UK and Japan noted reduced operations and treatments for gynaecological malignancies [30, 48]. In a tertiary gynecology centre in the UK, 2% of operations for gynecological malignancy were deferred or cancelled due to COVID-19, most often due to limited critical care and operating theatre capacity [69]. Additionally, participants in the UK, Canada, and Australia described difficulties accessing and delays in cervical cancer screening [14, 62, 70, 71].

Many institutions in the UK made changes to surgical care delivery including COVID-19 testing of patients, surgical team COVID-19 screening, weekly capacity reviews, eye protection, movement of operations to a different site, and strategies to decrease risk of aerosol-generating procedures [72]. These institutions also found significantly higher 30-day postoperative complication rates in 2020 compared with 2019, potentially due to transfer of care to new settings and decreased accessibility of primary care [72].

### Sexual health services

Sixteen studies reported that COVID-19 contributed to varying degrees of reduced access or use of available sexual health services. Studies in Australia found reduced STI consultations before lockdown compared to during lockdown [73] a move toward telehealth for testing services [62, 74]. Similarly, sexual health providers in Canada emphasised severe reductions in the availability of sexually transmitted and blood-borne infection testing due to government deployment of staff toward the COVID-19 response; thus, some clinics closed or adopted fewer hours [75]. This study reported the adoption of two models of care: 1) a “quick clinic” for asymptomatic patients to self-test for gonorrhoea and chlamydia at the clinic, but not for tests that require blood (e.g., HIV); and 2) a virtual clinic which replaced most in-person testing services, with few exceptions for high-risk clients; virtual clinics were seen as largely acceptable across participants [75].

Numerous studies reported reduced access or delays in receiving contraception care. One-quarter of adolescents and young adults in a Scottish study self-reported reduced access to and use of contraceptives and condoms due to the pandemic [76]. In an Italian study, 51.5% of participants reported that the pandemic adversely impacted procreative and contraceptive counselling services [12]. In Australia during the lockdown, 9% of women experienced increased difficulty accessing contraception, due to: lower stock of medication, challenges booking appointments for long-acting reversible contraception, less ability or desire to leave their residence due to COVID-19, their living situation, and fear of COVID-19 exposure [77]. Additionally, a UK study found that women had nine times greater likelihood of describing challenges accessing contraception after lockdown compared to before lockdown, and women conceiving after lockdown were more likely to have an unplanned pregnancy [78].

Some studies reported age, income, race, and gender identity differences in access to sexual health services. Access to sexual health services was lower among younger adults from 18 to 24 years old and unemployed women [77]. Similarly, a New Zealand study found that 22% of adolescents and young adults had unmet SRH needs during lockdown due to lack of information about service availability (41%) or COVID-19-related concerns (26%) [79]. A UK study found dramatic reductions in face-to-face sexual health service attendance for ages 13 and below (100% reduction), 14–15 (52% reduction), and 16–17 years old (31% reduction), suggesting that in-person services were a barrier to access during the pandemic especially for younger patients [80]. A Canadian study on university students found that cisgender women were two times more likely to report decreased access to STI testing/treatment and reproductive health services compared to cisgender men [81]. Other Canadian studies reported that Indigenous people identified greater difficulty accessing contraception than non-Indigenous Canadians [71] and decreased use of condoms among Two-Spirit, gay, bisexual and queer (2SGBQ+) men due to COVID-19 [82]. One study in Australia briefly reported that virtual care enabled could remove barriers to seeking out specialists for gender-affirming care [62].

### HIV care

Ten studies discussed the impact of the COVID-19 pandemic on people living with HIV. An Australia study reported the distribution of at-home HIV testing, although some patients were not enthusiastic about its use [62]. A Canadian study with participants who identified as 2SGBQ+ men, reported a 28% reduction in access to HIV testing due to COVID-19, particularly among individuals residing in more remote, country locations [82].

Three studies reported minimal changes in HIV medication management due to COVID-19 [83–85]. For example, an Australian study found that most participants had continuous access to their HIV-care provider, antiretroviral therapy, and monitoring tests [85]. Any barriers were due to transportation issues from lockdown measures, care provider cancellations, and social isolation [85].

Studies in Australia, Italy, Spain, and Belgium reported that there were no significant negative impacts of COVID-19 on virologic suppression [83, 84, 86], with one study finding a decreased number of patients experiencing virologic failure compared to before the pandemic [87]. This is despite finding decreased clinic visits, new consultations, laboratory tests, and timeliness of testing during the pandemic [74, 83, 84, 87]. In addition, a Canadian study found that cisgender women were 1.73 times more likely to report decreased access to HIV testing and treatment compared to cisgender men and students of colour were 1.54 times more likely to report decreased access to HIV services compared to white students [81].

To help reduce barriers to HIV medication management, Australian studies reported an increased proportion of HIV antiretroviral therapy sent to patients through home delivery [74, 84, 86], increased phone consultations and prescription sizes [74], and extended duration between appointments [62]. Virtual HIV care enabled some to access HIV specialist services despite living in remote areas [62]. Another study found a significant reduction in the mean weekly prescriptions of preexposure prophylaxis one week after restrictions were implemented, possibly due to reduced sexual behaviour or fewer visits to healthcare providers [88].

## Discussion

### Main findings

This systematic review synthesised 82 studies on the impact of COVID-19 on access and delivery of SRH services in high-income OECD countries with comparable economies and UHC systems. In this context, research on SRH is concentrated in relatively few countries: namely the UK, Australia, Italy, and Canada, and the changes made in other countries remains limited. Overall, considering the number of countries and breadth of SRH services included, there is a paucity of research studying the impact of COVID-19 on access to or delivery of SRH in UHC countries. Despite this, common themes were noted between countries and services that may be generalizable to these settings.

During the pandemic, many SRH services decreased capacity or ceased service provision altogether. As a result, many patients reported difficulty accessing care across SRH services while others avoided care to prevent COVID-19 exposure, both of which impact the provision of necessary care. Changes in response to the pandemic were heterogeneous across countries and services; in fact, there seems to be great variation even between institutions within countries. Multiple factors may have contributed to this variation: uncertainty with appropriate changes necessary to decrease COVID-19 transmission early in the pandemic, variations in geographical contexts, and differences in policy. In some studies, differences in practice were due to incomplete communication of policy decisions, with some healthcare providers not being aware of changes in guidance [26, 41]. Due to heterogeneity in study design and outcome measures, timelines, and healthcare system structure, it is challenging to directly compare countries. Rather, we draw on relevant general patterns and themes across the SRH spectrum.

Across outpatient services, providers favoured models of care that prevented patients from attending clinics, including virtual or telephone appointments, home monitoring or testing (e.g. blood pressure in antepartum care), sending prescriptions via postage, and increasing prescription sizes. Hospitals favoured models of care that reduced time and number of people in hospital and aerosol-generating environments. These included home IOL, postponing elective surgeries for those with COVID-19, decreasing the number of support people present during labour and postpartum, favouring neuraxial anaesthesia over general anaesthesia, and shifting care to outpatient or virtual clinics when possible (e.g. medical management of abortions and ectopic pregnancies). Some patients preferred to avoid healthcare institutions, favouring home births, for example, which often had fewer caregiver restrictions and exposures in hospitals. Altogether, this often resulted in decreased healthcare utilisation, delays in testing and treatment, and new gaps in SRH access. The relative implementation of these changes varied greatly by institution and country. For example, the UK Health Security Agency (UKHSA) reported lower numbers of consultations, sexual health screenings, and STI diagnoses in 2020 and 2021 as compared to previous years [89]. However, there were also local and regional differences in chlamydia detection due to testing coverage, variation in settings that offered testing, and variations in COVID-19 disruptions [89]. Similarly in Canada, The Public Health Agency of Canada reported decreases in STBBI prevention, testing, and treatment services, as well as decrease in staffing during the first year of the pandemic [90].

These changes are also not unique to SRH. For example, Moynihan et al. (2021) [91] showed decreased overall healthcare utilisation by one third across 20 countries. Studies have reported delays and disruptions in the provision of cancer services and cancer surgery due to the pandemic [92, 93]. The long-term impact of COVID-19 on decreased access to SRH is not yet fully understood. Despite delays in testing and decreased access to care, studies in Italy, Belgium, and Australia mentioned viral suppression for patients with HIV were not affected [83, 84, 87]. Rimmer et al. described reduced oncology operating capacity as alarming with potentially increased morbidity in the long-term. For other services, long-term outcomes were not available [30]. However, a systematic review and meta-analysis by Chmielewska et al. reported increased mortality among pregnant women, stillbirth, ruptured ectopic pregnancies, and peripartum depression during the COVID-19 pandemic across 17 countries [94]. To better understand the impacts of these changes and influences on future policy, additional research on the long-term impacts of the pandemic on SRH is necessary.

This review found a limited number of studies that report on the impact of COVID-19 on SRH among underserved populations, such as sexual and gender minorities, people of low socioeconomic status, rural communities, racial/ethnic minorities, and people living with disabilities. Given the exacerbation of inequities created by the pandemic and reduced access to certain services, further research should focus on these populations to determine whether COVID-19-related adaptations and innovations have equitable impacts, especially as it relates to these populations.

### Limitations

This study has limitations. While this review was restricted to high-income countries with UHC, there were many underrepresented countries, and the results of this review may not generalise even within this subset. Many smaller changes across institutions may not have been reported in the published literature, instead being implemented and understood through institutional policies (e.g. increased use of PPE). Finally, most studies included were of lower levels of evidence, being observational in nature. To improve the robustness of this review, only studies with a quantitative or qualitative comparator to a previous time period or prior experience were included to better isolate the impact of the pandemic.

## Conclusion

In conclusion, the COVID-19 pandemic has had significant and heterogeneous impacts on a wide range of SRH services across high-income OECD countries with UHC, typically resulting in decreased access to and delivery of care. Studies were concentrated in relatively few countries. The long-term impacts of these changes and the impact of these changes on underserved populations remains uncharacterized and future research is required in these areas, especially in countries with less current information available. In addition, future studies should examine the impact of sustained service changes that were initially made during COVID-19 that could have benefits beyond the pandemic.

## Supporting information

S1 Checklist PRISMA 2009 checklist. (DOCX)

S1 Fig PRISMA diagram. (TIF)

S1 Table List of universal health care countries included in review. (TIF)

S2 Table Quality assessment. (DOCX)
