# Supplementary figures and images for: Impact of COVID-19 on access to and delivery of sexual and reproductive healthcare services in countries with universal healthcare systems: A systematic review
Source: PLoS One. 2024 Feb 23;19(2):e0294744. doi: 10.1371/journal.pone.0294744 (PMC10889625; doi:10.1371/journal.pone.0294744)

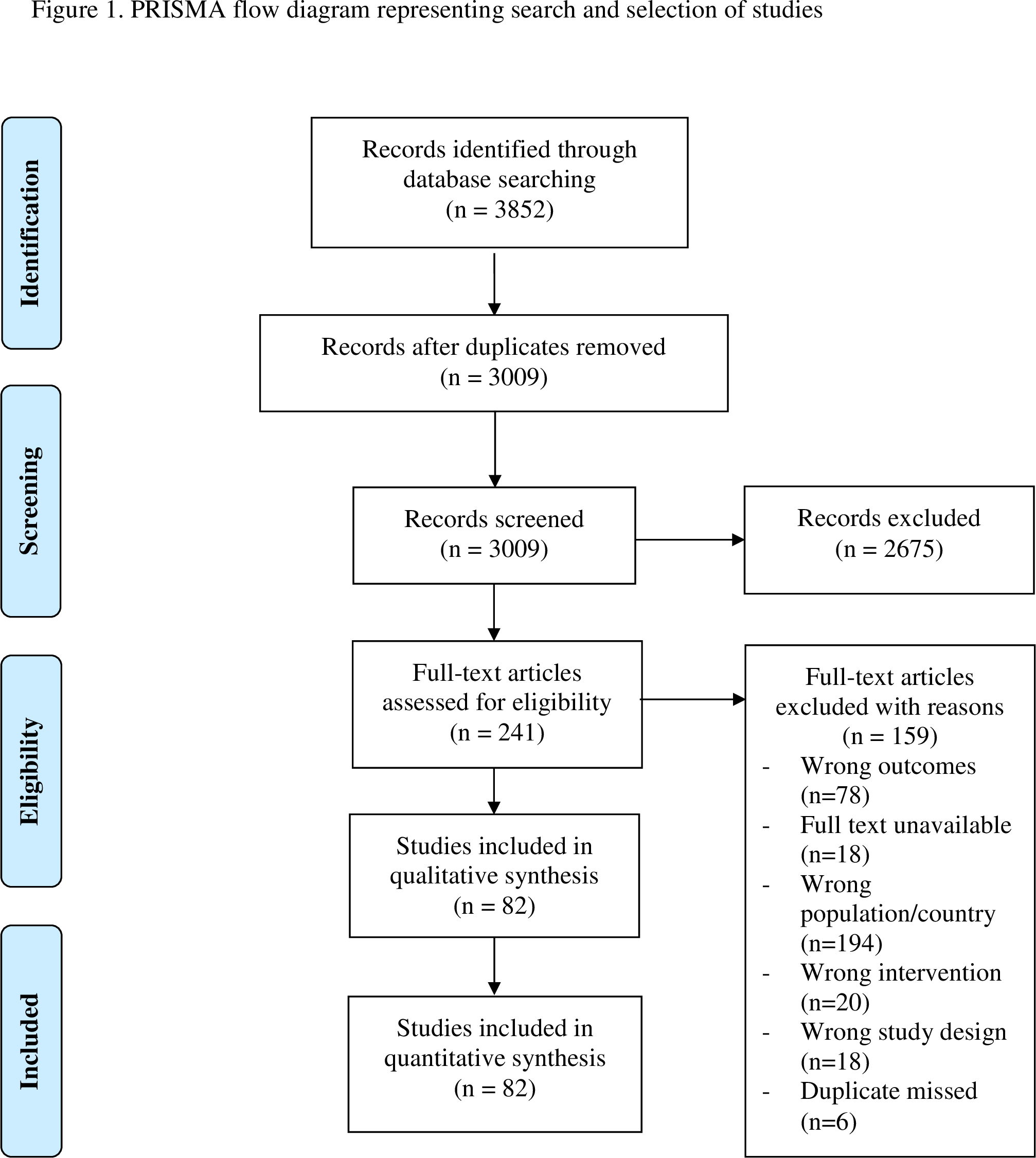

Supplement: S1 Fig — (TIF) [file pone.0294744.s002.tif]

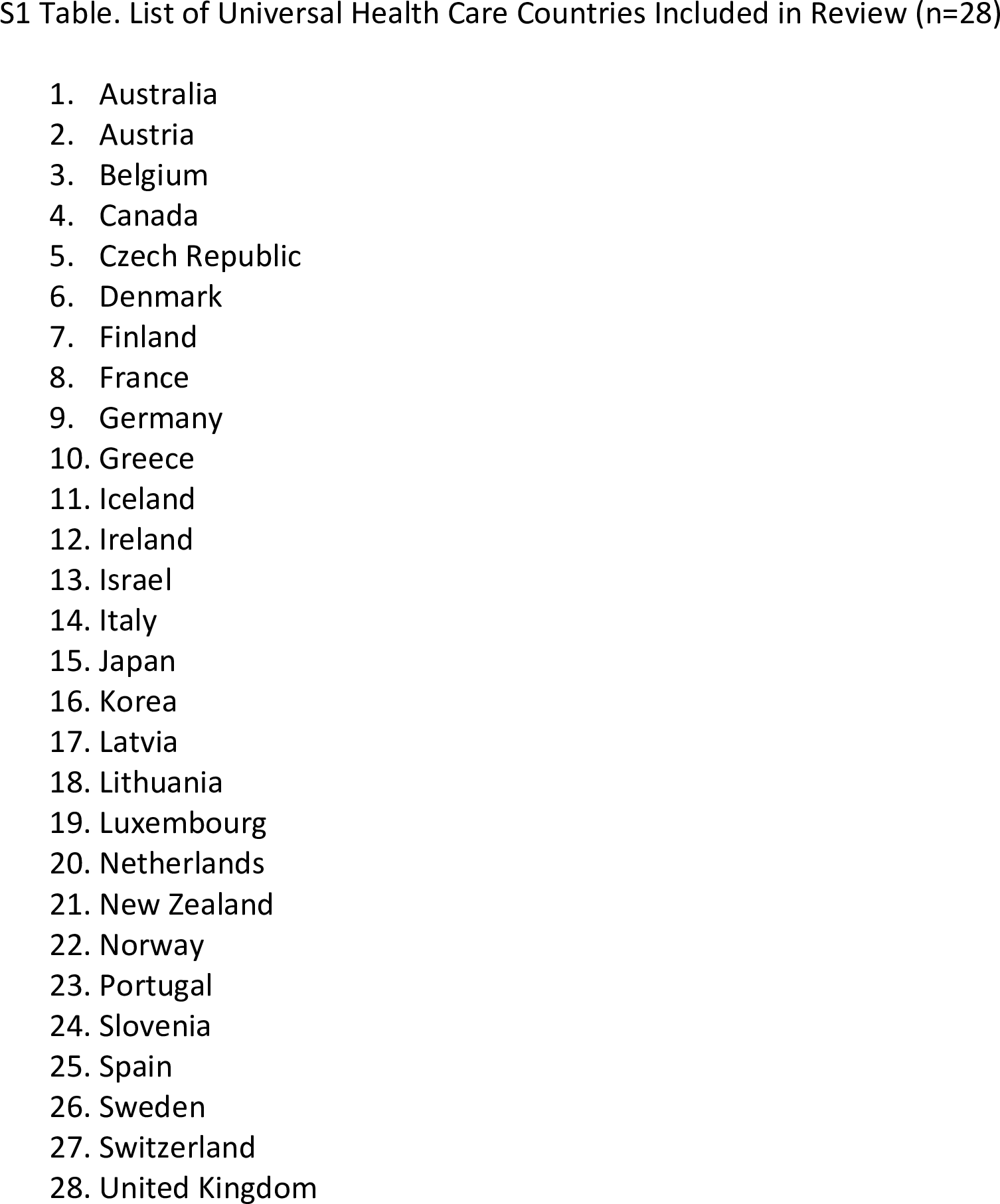

Supplement: S1 Table — (TIF) [file pone.0294744.s003.tif]
