# Supplementary material for: Impact of COVID-19 on access to and delivery of sexual and reproductive healthcare services in countries with universal healthcare systems: A systematic review
Source: PLoS One. 2024 Feb 23;19(2):e0294744. doi: 10.1371/journal.pone.0294744 (PMC10889625; doi:10.1371/journal.pone.0294744)
Supplement: S2 Table — (DOCX) [file pone.0294744.s004.docx]

S2 Table. Quality Assessment

| **Reference** | **C1** | **C2** | **C3** | **C4** | **C5** | **C6** | **C7** | **C8** | **C9** | **C10** | **C11** | **C12** | **C13** | **Score** |
| --- | --- | --- | --- | --- | --- | --- | --- | --- | --- | --- | --- | --- | --- | --- |
| Atay et al. | 3 | 2 | 2 | 3 | 2 | 2 | 2 | 2 | 2 | 2 | 3 | 0 | 2 | 27 |
| Aydin et al. | 3 | 3 | 3 | 2 | 2 | 2 | 2 | 3 | 3 | 2 | 3 | 0 | 2 | 30 |
| Baaske et al. | 1 | 2 | 2 | 2 | 2 | 1 | 2 | 2 | 2 | 1 | 2 | 0 | 2 | 21 |
| Balachandren et al. | 2 | 3 | 3 | 2 | 3 | 3 | 3 | 2 | 3 | 2 | 3 | 0 | 2 | 31 |
| Baravelli et al. | 2 | 3 | 3 | 3 | 3 | 3 | 3 | 2 | 1 | 3 | 3 | 0 | 3 | 32 |
| Bayrampour et al. | 1 | 3 | 3 | 2 | 2 | 2 | 2 | 3 | 3 | 3 | 2 | 0 | 2 | 28 |
| Bekaert et al. | 3 | 3 | 1 | 2 | 1 | 1 | 1 | 1 | 2 | 1 | 2 | 0 | 1 | 19 |
| Binyamin et al. | 2 | 2 | 3 | 3 | 2 | 2 | 3 | 2 | 2 | 1 | 2 | 0 | 1 | 25 |
| Bittleston et al. | 3 | 3 | 3 | 2 | 1 | 2 | 2 | 3 | 2 | 1 | 2 | 0 | 1 | 25 |
| Boisvert et al. | 2 | 2 | 2 | 2 | 2 | 1 | 3 | 2 | 3 | 2 | 2 | 1 | 2 | 26 |
| Boso et al. | 2 | 3 | 2 | 3 | 2 | 2 | 3 | 3 | 2 | 2 | 3 | 0 | 3 | 30 |
| Bradfield et al. | 2 | 2 | 2 | 3 | 2 | 3 | 3 | 3 | 3 | 3 | 2 | 2 | 2 | 32 |
| Brandell et al. | 1 | 2 | 3 | 2 | 2 | 2 | 2 | 1 | 2 | 1 | 2 | 0 | 2 | 20 |
| Cameron et al. | 3 | 3 | 2 | 3 | 2 | 3 | 3 | 3 | 2 | 3 | 3 | 0 | 2 | 32 |
| Cena et al. | 1 | 2 | 2 | 2 | 2 | 3 | 3 | 2 | 2 | 2 | 2 | 2 | 2 | 27 |
| Ceulemans et al. | 3 | 3 | 3 | 2 | 2 | 3 | 2 | 2 | 3 | 2 | 3 | 1 | 3 | 32 |
| Chow et al. | 2 | 3 | 3 | 2 | 2 | 3 | 3 | 3 | 3 | 2 | 3 | 0 | 2 | 31 |
| Clark et al. | 2 | 3 | 3 | 3 | 2 | 3 | 2 | 3 | 2 | 1 | 1 | 0 | 3 | 28 |
| Coombe et al. | 2 | 2 | 3 | 2 | 1 | 2 | 2 | 2 | 3 | 2 | 3 | 0 | 2 | 26 |
| Corrao et al. | 3 | 3 | 2 | 3 | 2 | 2 | 2 | 1 | 1 | 3 | 2 | 0 | 2 | 26 |
| Cruz-Ramos et al. | 2 | 2 | 2 | 2 | 1 | 1 | 1 | 2 | 2 | 2 | 2 | 1 | 1 | 21 |
| Cui et al. | 2 | 2 | 2 | 2 | 2 | 2 | 2 | 2 | 1 | 2 | 3 | 0 | 2 | 24 |
| De kort et al. | 3 | 3 | 3 | 3 | 2 | 2 | 3 | 2 | 3 | 1 | 2 | 0 | 2 | 29 |
| Dell'Utri et al. | 2 | 3 | 3 | 2 | 2 | 2 | 2 | 2 | 2 | 1 | 2 | 0 | 1 | 24 |
| Dixon et al. | 2 | 2 | 3 | 2 | 2 | 2 | 3 | 2 | 3 | 2 | 2 | 0 | 1 | 26 |
| Doncarli et al. | 3 | 3 | 2 | 2 | 3 | 1 | 2 | 3 | 3 | 2 | 3 | 0 | 2 | 29 |
| Dorizzi et al. | 1 | 2 | 2 | 2 | 0 | 2 | 2 | 2 | 1 | 1 | 1 | 2 | 0 | 18 |
| El Moussaoui et al. | 3 | 2 | 2 | 2 | 2 | 1 | 2 | 2 | 3 | 2 | 2 | 0 | 1 | 24 |
| Ennis et al. | 3 | 3 | 2 | 3 | 1 | 1 | 3 | 2 | 2 | 1 | 2 | 1 | 1 | 25 |
| Fletcher et al. | 2 | 3 | 3 | 3 | 2 | 3 | 3 | 3 | 3 | 1 | 2 | 2 | 2 | 32 |
| Gamberini et al. | 2 | 3 | 3 | 3 | 2 | 2 | 3 | 2 | 2 | 2 | 2 | 1 | 2 | 29 |
| Giacomelli et al. | 2 | 3 | 3 | 3 | 2 | 2 | 3 | 3 | 1 | 2 | 3 | 0 | 2 | 29 |
| Gomez et al. | 2 | 3 | 3 | 3 | 2 | 2 | 3 | 3 | 2 | 2 | 3 | 0 | 2 | 30 |
| Gonzalez-Timoneda et al. | 3 | 3 | 3 | 3 | 3 | 2 | 2 | 3 | 2 | 3 | 3 | 0 | 2 | 32 |
| Greene et al. | 2 | 3 | 2 | 2 | 1 | 2 | 1 | 2 | 2 | 2 | 2 | 0 | 2 | 23 |
| Harkness et al. | 3 | 3 | 3 | 2 | 3 | 3 | 2 | 2 | 3 | 3 | 3 | 0 | 0 | 30 |
| Henry et al. | 3 | 3 | 3 | 3 | 2 | 1 | 2 | 3 | 2 | 1 | 2 | 0 | 2 | 27 |
| Hertlea et al. | 2 | 2 | 2 | 2 | 1 | 3 | 2 | 3 | 3 | 0 | 1 | 2 | 2 | 25 |
| Herzberger et al. | 1 | 3 | 2 | 2 | 3 | 2 | 2 | 3 | 2 | 2 | 2 | 0 | 2 | 26 |
| Homer et al. | 3 | 2 | 3 | 2 | 2 | 1 | 2 | 2 | 2 | 1 | 2 | 0 | 1 | 23 |
| Hukku et al. | 3 | 3 | 2 | 2 | 2 | 3 | 3 | 3 | 2 | 2 | 2 | 0 | 2 | 29 |
| Jardine et al. | 2 | 3 | 3 | 2 | 3 | 2 | 2 | 2 | 3 | 2 | 2 | 1 | 2 | 29 |
| John et al. | 2 | 3 | 3 | 3 | 2 | 1 | 2 | 2 | 2 | 1 | 2 | 1 | 3 | 27 |
| Justman et al. | 2 | 2 | 3 | 3 | 2 | 1 | 2 | 2 | 2 | 2 | 2 | 0 | 2 | 25 |
| Karavani et al. | 1 | 2 | 2 | 2 | 2 | 2 | 2 | 2 | 2 | 2 | 2 | 0 | 1 | 22 |
| Khourya et al. | 3 | 3 | 3 | 2 | 2 | 3 | 3 | 2 | 3 | 2 | 3 | 0 | 3 | 32 |
| Kluwgant et al. | 3 | 3 | 2 | 2 | 1 | 2 | 2 | 1 | 2 | 1 | 2 | 3 | 2 | 26 |
| Komatsu et al. | 1 | 1 | 2 | 2 | 1 | 1 | 2 | 1 | 1 | 0 | 2 | 0 | 0 | 14 |
| Lam et al. | 2 | 3 | 3 | 2 | 2 | 2 | 2 | 2 | 2 | 2 | 2 | 0 | 1 | 25 |
| Lee et al. | 3 | 3 | 3 | 2 | 2 | 2 | 2 | 3 | 2 | 3 | 2 | 0 | 3 | 30 |
| Leung et al. | 3 | 3 | 3 | 3 | 2 | 1 | 3 | 2 | 3 | 1 | 2 | 0 | 3 | 29 |
| Lewis et al. | 3 | 3 | 3 | 2 | 2 | 1 | 2 | 2 | 2 | 1 | 2 | 3 | 1 | 27 |
| Lowe-Zinolaa et al. | 2 | 2 | 2 | 2 | 1 | 2 | 2 | 2 | 2 | 1 | 2 | 0 | 1 | 21 |
| Ma et al. | 2 | 3 | 3 | 2 | 1 | 2 | 2 | 2 | 2 | 1 | 1 | 0 | 3 | 24 |
| Moltrecht et al. | 3 | 3 | 3 | 2 | 1 | 2 | 2 | 3 | 2 | 2 | 2 | 0 | 3 | 28 |
| Montgomerya et al. | 2 | 2 | 3 | 3 | 2 | 2 | 2 | 2 | 2 | 3 | 3 | 0 | 2 | 28 |
| Munda et al. | 1 | 2 | 3 | 2 | 2 | 2 | 3 | 3 | 2 | 2 | 2 | 0 | 2 | 26 |
| Nakagawa et al. | 3 | 2 | 3 | 2 | 2 | 1 | 2 | 2 | 2 | 1 | 3 | 0 | 1 | 24 |
| Newman et al. | 2 | 2 | 1 | 2 | 1 | 1 | 2 | 2 | 1 | 2 | 2 | 0 | 0 | 18 |
| Overbeck et al. | 2 | 3 | 3 | 2 | 1 | 1 | 2 | 2 | 2 | 1 | 1 | 2 | 1 | 23 |
| Phillips et al. | 3 | 3 | 3 | 2 | 3 | 1 | 3 | 3 | 2 | 1 | 1 | 1 | 1 | 27 |
| Potenza et al. | 2 | 2 | 2 | 1 | 1 | 1 | 1 | 2 | 1 | 1 | 2 | 0 | 0 | 16 |
| Quirós-Gonzáleza et al. | 2 | 2 | 3 | 2 | 2 | 2 | 2 | 3 | 2 | 2 | 2 | 0 | 2 | 26 |
| Riley et al. | 2 | 2 | 2 | 2 | 1 | 2 | 2 | 3 | 1 | 2 | 2 | 0 | 1 | 22 |
| Rimmer et al. | 1 | 2 | 2 | 2 | 3 | 1 | 2 | 2 | 2 | 2 | 2 | 1 | 1 | 23 |
| Rød et al. | 3 | 3 | 3 | 3 | 2 | 2 | 2 | 3 | 3 | 1 | 2 | 0 | 2 | 29 |
| Rose et al. | 2 | 3 | 3 | 2 | 2 | 2 | 2 | 3 | 2 | 1 | 2 | 2 | 3 | 29 |
| Ryu et al. | 3 | 3 | 2 | 3 | 1 | 2 | 2 | 2 | 2 | 2 | 2 | 1 | 2 | 27 |
| Sarre et al. | 3 | 3 | 3 | 2 | 2 | 2 | 2 | 2 | 2 | 1 | 2 | 0 | 1 | 25 |
| Schaler et al. | 1 | 2 | 3 | 2 | 1 | 0 | 2 | 2 | 2 | 1 | 2 | 0 | 2 | 20 |
| Silverio et al. | 2 | 2 | 3 | 3 | 2 | 3 | 3 | 3 | 2 | 3 | 3 | 0 | 2 | 31 |
| Souleymanov et al. | 3 | 3 | 3 | 2 | 2 | 2 | 3 | 2 | 2 | 2 | 2 | 3 | 2 | 31 |
| Suárez-Cortés et al. | 2 | 2 | 2 | 2 | 1 | 2 | 2 | 2 | 2 | 3 | 2 | 1 | 2 | 25 |
| Sweet et al. | 3 | 2 | 2 | 3 | 3 | 1 | 2 | 3 | 3 | 2 | 3 | 0 | 1 | 28 |
| Traeger et al. | 2 | 1 | 2 | 2 | 2 | 1 | 2 | 1 | 1 | 1 | 2 | 0 | 0 | 17 |
| Trinh et al. | 2 | 3 | 3 | 2 | 2 | 1 | 3 | 3 | 3 | 2 | 1 | 0 | 2 | 27 |
| Vitabile et al. | 1 | 3 | 3 | 2 | 2 | 2 | 2 | 3 | 3 | 1 | 2 | 0 | 1 | 25 |
| Weerasuria et al. | 1 | 2 | 2 | 2 | 1 | 2 | 1 | 2 | 2 | 0 | 1 | 2 | 2 | 20 |
| Wilson et al. | 2 | 2 | 2 | 2 | 2 | 2 | 2 | 2 | 2 | 1 | 2 | 0 | 1 | 22 |
| Wilson et al. | 2 | 2 | 2 | 2 | 2 | 2 | 2 | 2 | 2 | 1 | 2 | 1 | 2 | 24 |
| Wood et al. | 3 | 3 | 3 | 2 | 2 | 3 | 3 | 2 | 3 | 2 | 2 | 1 | 2 | 31 |
| Zaighama et al. | 2 | 2 | 1 | 1 | 1 | 2 | 2 | 2 | 3 | 2 | 3 | 1 | 2 | 24 |
